# Control charts for monitoring mood stability as a predictor of severe episodes in patients with bipolar disorder

**DOI:** 10.1186/s40345-017-0116-2

**Published:** 2018-04-04

**Authors:** Maria D. L. A. Vazquez-Montes, Richard Stevens, Rafael Perera, Kate Saunders, John R. Geddes

**Affiliations:** 1 Nuffield Department of Primary Care Health Sciences, University of Oxford, Radcliffe Observatory Quarter, Woodstock Road, Oxford, OX2 6GG UK; 2 Department of Psychiatry, Warneford Hospital, University of Oxford, Warneford Lane, Oxford, OX3 7JX UK

**Keywords:** Bipolar disorder, Episode prediction, Mood variability, Control charts, Shewhart’s control rules, Sensitivity, Positive predictive value

## Abstract

**Background:**

Recurrent mood episodes and subsyndromal mood instability cause substantial disability in patients with bipolar disorder. Early identification of mood episodes enabling timely mood stabilization is an important clinical goal. This study investigates the ability of control chart methodology to predict manic and/or depressive episodes by applying Shewhart’s control rules to weekly self-reported scores from mania and depression questionnaires.

**Methods:**

Shewhart’s control rules were applied to weekly self-reported scores from the Altman Self-Rating Mania Scale (ASRM) and the Quick Inventory of Depressive Symptomatology—Self-Report (QIDS) collected from 2001 to 2012 as part of the OXTEXT programme. Manic and depressive episodes were defined as an ASRM score ≥ 10 or a QIDS score ≥ 15, respectively. An episode-free run-in period of eight consecutive weeks without an episode of either type was used to calibrate control charts. Shewhart’s rules were then applied to follow-up data. Their sensitivity and positive predictive value for predicting manic or depressive episodes within the next 4 weeks were calculated focusing on the first episode. Secondary analyses varying control chart type, length of episode-free run-in period, time frames to evaluate diagnostic accuracy, thresholds defining either manic or depressive episodes, and missing data methods were performed.

**Results:**

Data from 146 participants (37% men) were included. The mean age was 43.4 (SD = 13.3) years. The median follow-up was 10 (IQR 5–40) weeks for mania and 10 (IQR 5–23) weeks for depression. A total of 53 (36%) participants had a manic episode and 67 (46%) had a depressive episode. For manic episodes, the sensitivity and positive predictive value of Shewhart’s control rules were 30% (95% CI 19–45%) and 7% (95% CI 5–9%), and for depressive episodes, 33% (95% CI 22–46%) and 9% (95% CI 6–12%), respectively. Results from secondary analyses were similar to these.

**Conclusions:**

Tele-monitoring with control rules has the potential to predict about one-third of manic or depressive episodes before they occur, at the cost of a high false positive rate. Given the severe consequences of manic and depressive episodes, this trade-off may be desirable.

**Electronic supplementary material:**

The online version of this article (10.1186/s40345-017-0116-2) contains supplementary material, which is available to authorized users.

## Background

Bipolar disorder is a mental illness characterized by recurrent manic and depressive symptoms occurring as both acute episodes and subsyndromal mood instability. It has a prevalence of approximately 1–2% in the general population (Mayora et al. 2013) and is the mental disorder with the highest suicide rate (Hawton et al. 2005). Recurrent episodes and mood instability cause substantial disability and so prevention of severe episodes and mood stabilization are important therapeutic targets.

Conventional treatments for bipolar disorder include a combination of drugs [lithium being the most commonly used (Geddes et al. 2004)] and psychotherapy (Geddes et al. 2013). As a means to prevent relapse, current treatment research is focusing on understanding mood variability (Bonsall et al. 2012). Efforts have been directed towards development of technology for continuous monitoring of more objective parameters to aid treatment (e.g. voice frequency readers, wrist-worn activity monitors, mobile electro-dermal activity sensors) (Mayora et al. 2013). The use of text messaging and email has been implemented for weekly self-report of mood scores, for instance by the OXTEXT programme (https://oxfordhealth.truecolours.nhs.uk/www/en/; Bopp et al. 2010). Such approaches are beneficial as retrospective accounts are inherently unreliable, unable to measure temporal variation or to identify mood instability that is mild but nevertheless functionally significant. Remote monitoring methodologies are a mechanism to reduce this recall bias and give us greater insight into the dynamic nature of mood instability in daily life.

Prediction models for episodes have so far considered covariates correlated with symptom severity, treatment complexity, or remission to predict long-term outcomes (Busch et al. 2012). However, variability between severe episodes has not been considered as a potential predictor. Control charts are a tool focused on the variability of a process which can potentially be used to identify when an episode is about to occur.

Control charts are widely used in industry. They are a visual display of a process over time combined with algorithms called ‘control rules’ designed to distinguish systematic change in the underlying process from random noise. A run-in period of data collected under stability informs the user of the inherent variability of the process. This variability is used to calculate control limits usually as 1, 2, and 3 deviations from the mean. Control charts are subsequently applied to prospectively monitor the process stability and control. Their application in medicine has expanded in recent years (Thor et al. 2007; Mohammed et al. 2008) in topics as varied as maintaining quality of electronic medical records (Siregar et al. 2013), evaluating performance in cardiac surgery (Smith et al. 2013), or managing patients with asthma (Alemi and Neuhauser 2004). They have been shown to be effective tools in the management of other medical conditions. In particular, they are useful for immediate detection of unusual change allowing for early intervention and prevention (Smith et al. 2013).

This study investigates the ability of the control chart methodology to predict manic or depressive episodes in patients with bipolar disorder by applying Shewhart’s control rules to weekly self-reported scores from mania and depression self-measurement questionnaires. The main analysis considers control charts based on mean and standard deviation across all patients’ episode-free run-in periods. The sensitivity and positive predictive value (PPV) of Shewhart’s control rules for predicting manic or depressive episodes within the next 4 weeks are reported. Secondary analyses using a longer episode-free run-in period, different control chart types, different time frames to evaluate diagnostic accuracy parameters, lower thresholds defining episodes of either type, and missing data methods were also performed.

## Methods

### Data

The OXTEXT programme, funded by the UK National Institute of Health Research, investigates the benefit of self-monitoring in people with bipolar disorder using the True Colours self-management system (https://oxfordhealth.truecolours.nhs.uk/www/en/). Participants in the programme were prompted weekly by text messages or email to complete and return self-measurement questionnaires.

Mania was assessed using the Altman Self-Rating Mania Scale (ASRM) (Altman et al. 1997). This scale is formed of five items evaluating mood, self-confidence, sleep disturbance, speech, and activity level over the past week. Each item can take a value between 0 and 4. The total score for this scale ranges from 0 to 20. Higher scores indicate higher manic mood severity. A manic episode was defined as any instance where the ASRM score was greater or equal to 10, which is a pragmatic cut point to reflect clinically significant mania observed in the wider OXTEXT cohort (judged by OXTEXT programme experts).

Depression is assessed using the Quick Inventory of Depressive Symptomatology—Self Report (QIDS) (Rush et al. 2000). This scale is formed of 16 items evaluating nine symptom domains for depression according to DSM-IV (Association AP 1994) in the past week: sad mood, concentration, self-criticism, suicidal ideation, interest, energy/fatigue, sleep disturbance (initial, middle, and late insomnia or hypersomnia), decrease or increase in appetite or weight, and psychomotor agitation or retardation. Each domain can take a value between 0 and 3. The total score for this scale ranges from 0 to 27. Higher scores indicate higher severity of depression. A depressive episode was defined as any instance where the QIDS score was greater or equal to 15, which is the cut point defined as that for severe depression (Rush et al. 2003; University of Pittsburgh Epidemiology Data Centre 2017).

**Fig. 1:**
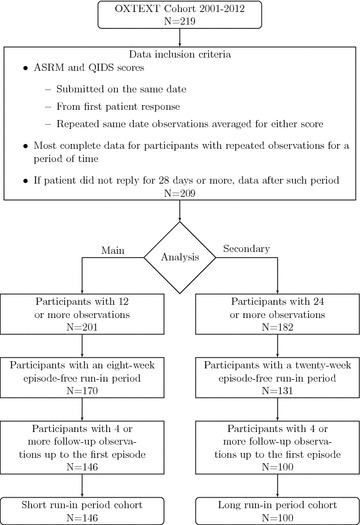
Flow diagram of data inclusion. Data were selected from the starting OXTEXT cohort 2001–2012 according to inclusion criteria designed to reduce missing data. Cohorts for the main and secondary analyses were then selected based on the number of available individual observations such that every participant included had a period of eight consecutive weeks without either a manic or depressive episode (episode-free run-in period) and at least four follow-up observations up to the first episode

Data inclusion criteria were based on ASRM and QIDS scores submitted on the same date. Initial non-responses, assumed to be a participant’s training period, were excluded. When a participant submitted several responses for a particular scale at a given time point, the average of these observations was included in the analysis. When repeated measurements occurred for a period of time, the most complete data were selected for that period. For participants who had a gap of 28 days or more since the last observation, data from the first record after the gap were selected. After this initial data selection, only participants with 12 or more records were considered, including only those who had a period of eight consecutive weeks without either a severe mania or depression episode (see below the definition of mania and depression episodes) after which period data were limited up to the first severe episode. Figure 1 gives a schematic representation of this inclusion/exclusion process. We will refer to the retained sample as the “short run-in period cohort”.

### Control charts

For each of ASRM and QIDS, we developed four types of control charts, based on the same dataset: X-bar charts, personalized X-bar charts, individual-moving range charts, and run sum charts. An episode-free run-in period was defined as the first eight consecutive weeks without an episode of either type. A minimum of 50% observed values during the 8 weeks was required to establish the episode-free run-in period for each scale.

**Fig. 2:**
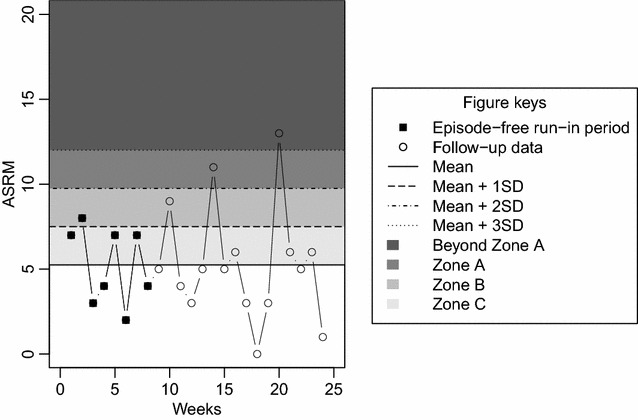
Control chart and control chart zones. A control chart is a graphical representation of a process over time, for instance weekly ASRM scores, where control limits defined by the number of standard deviations from the mean are highlighted. The areas between the control limits define control zones used to subsequently apply control rules that will allow the user to identify observations potentially showing a special cause variability and not simply random variability. This study considered only control zones above the mean, because in the context of bipolar disorder unusually high mood scores are of greater interest than low ones. This figure shows an X-bar chart for randomly generated ARSM scores (mean = 5, SD = 3.3)

X-bar charts (Shewhart 1931) assume an underlying normal distribution of the data to define control limits as follows. The mean and standard deviation (SD) of the scores over the episode-free run-in period was calculated for all participants. The average, across all participants, of these means and SDs were subsequently used as global mean and SD to calculate universal upper control limits as the global mean plus one, two, or three global SDs. Lower control limits were not considered given that the medical interest in the context of bipolar disorder lies in detecting unusually high values, referred to as out-of-control values in the quality control literature (Montgomery 2013). To construct personalized X-bar charts, the control limits were calculated for each participant based on his/her mean score and SD over his/her episode-free run-in period. Figure 2 shows an example of an X-bar control chart for randomly generated ARSM data (mean = 5, SD = 3.3 points).

Individual-moving range charts are recommended in industry when single measurements are collected at a given time (Levinson 2010) as it happens with the monitoring of bipolar disorder patients. They are a pair of charts in which the first one displays the individual observations, with control limits based on their average and standard deviation estimated using the average moving range of pairs of consecutive measurements. The second chart displays the moving ranges, with control limits calculated using the average moving range and its estimated standard deviation. The function *qcc()* in R can be used to automatically calculate the limits and control charts themselves.

Run sum charts (Reynolds 1971; Aguirre-Torres and Reyes-López 1999) are also a pair of personalized charts, named X-bar and R charts, used to assess changes in the mean and dispersion of the process due to special causes. These charts do not use Shewhart’s control rules separately. Instead, they incorporate a simple automatic procedure in which weights previously assigned to each control chart zone are added up depending on where the data, sampled beforehand, fall. Starting at zero, weights are added for all observations on the same side of the central line of the chart. Once an observation falls on the opposite side of the central line, the sum is reset to the weight associated with the control zone where the observation fell. The latter is also done when the total sum of weights exceeds a pre-specified threshold. In such case, a special variation cause is identified at the point where the threshold was overcome. In this work, Reynolds’ (1971) weights and threshold were considered. These are: 0 for observations in Zone C, 1 for observations in Zone B, 2 for observations in Zone A, and 3 for observations beyond Zone A (control zones are represented in Fig. 2 and explained in more detail in the next section); threshold 5. Aguirre-Torres and Reyes-López (1999) demonstrated that this score system is less sensitive to small changes in dispersion and is likely to reduce the amount of false alarms. They also introduced a procedure to construct the R chart that allows using the same score system in both run sum charts. We followed the procedure to construct the charts described in detail in Aguirre-Torres and Reyes-López (1999). Both run sum charts are based on the average and range of rational subgroups which are groups of successive observations from the same patient. We selected the minimum possible rational subgroup size that these charts allow: two observations. Because the run sum procedure can only be applied when data are observed, we assessed these charts on the following scenarios: (1) only available data are used, ignoring episode-free run-in periods; (2) all data used, with missing data imputed through last value carried forward procedure and ignoring episode-free run-in periods; (3) episode-free run-in periods used to define the control zones, using mean imputation if data missing, and the run sum procedure was carried out over follow-up data (a) available, and (b) imputed using last value carried forward. The charts were considered independently and jointly.

### Control limits and rules

All control charts were divided into zones covering the area above the mean (see Fig. 2), except for run sum charts that consider the area below the mean too. A measurement was defined to be: ‘beyond zone A’ if it lay more than 3 SD values above the mean (or 3SD values below the mean); ‘in zone A’ if it lay between 2 and 3 SD values above the mean (or between 2 and 3 SD values below the mean); ‘in zone B’ if it lay between 1 and 2 SD values above the mean (or between 1 and 2 SD values below the mean); ‘in zone C’ if it lay up to 1 SD above the mean (or up to 1 SD below the mean).

**Fig. 3:**
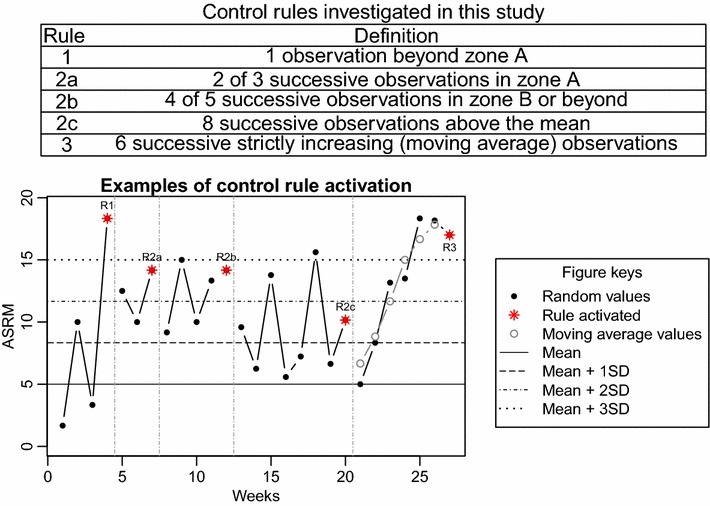
Control rules investigated in this study and examples of control rule activation. ASRM scores were randomly generated with mean = 5 and SD = 3.3. Independent sections where each of the five control rules investigated in this study was activated are shown. A control rule was said to be activated on the observation by which all its conditions were satisfied

This study looked individually at the five control rules listed in Fig. 3 and also at the case when any of the five rules was activated. For its generality, the results presented in this manuscript are based on the ‘any rule’ context. Results for the individual rules are shown in Additional files 1 and 2 for universal and personalized X-bar charts; and Additional files 3 and 4 for individual-moving range charts. Rules 1, 2a, 2b, and 2c are the usual first four Shewhart’s rules (Kane 1989). Shewhart’s rule 3 (6 successive increasing observations above the mean or 6 successive decreasing observations below the mean) was modified to be six successive strictly increasing observations above the mean, where the observations were transformed to the moving average of each observation and the observations on either side. A control rule was said to be activated on the observation by which all its conditions were satisfied. Figure 3 also shows examples of rule activation for randomly generated ASRM scores with mean = 5 and SD = 3.3 points.

**Fig. 4:**
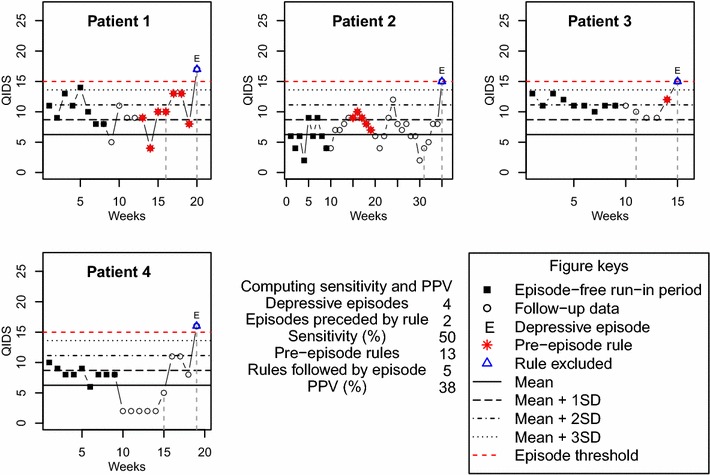
Example of sensitivity and positive predictive value calculation. Calculation of the sensitivity and positive predictive value of Shewhart’s control rules to predict a depressive episode in the next 4 weeks using universal X-bar charts (with mean = 6.2 and SD = 2.5) across a sample of four anonymized patients. QIDS data used in this plot have been imputed using last value carried forward

For each of ASRM and QIDS, we calculated the sensitivity and positive predictive value of Shewhart’s control rules for predicting manic or depressive episodes as follows. The sensitivity of control rule methodology for predicting clinical episodes was defined as the percentage of episodes that were preceded by activation of a rule in the previous 4 weeks across all participants. The positive predictive value (PPV) of control rule methodology was defined as the percentage of control rule activations that were succeeded by an episode within the next 4 weeks across all participants. To avoid introducing bias, activated rules that coincided with an episode were excluded from the analysis of PPV. Figure 4 shows an example in which the sensitivity and PPV of Shewhart’s control rules to predict depressive episodes in the next 4 weeks based on ‘any rule’ and using universal X-bar charts (with mean = 6.2 and SD = 2.5) are calculated. For this example, a random sample of four anonymized patients was extracted from the QIDS data imputed using last value carried forward (see “Secondary analyses” for details regarding this dataset). All depressive episodes and points where a rule was activated are highlighted differentiating between rules pre-episode and rules coinciding with an episode. Focusing on the first episode ever, the window of 4 weeks pre-episode to assess sensitivity is also indicated. In this example, there were a total of four depressive episodes, two of which were preceded by the activation of a rule in the previous 4 weeks. This gives a sensitivity of 50%. As for PPV, a total of 13 pre-episode rules were activated, 5 of which were succeeded by an episode in the next 4 weeks, giving a PPV of 38%.

**Fig. 5:**
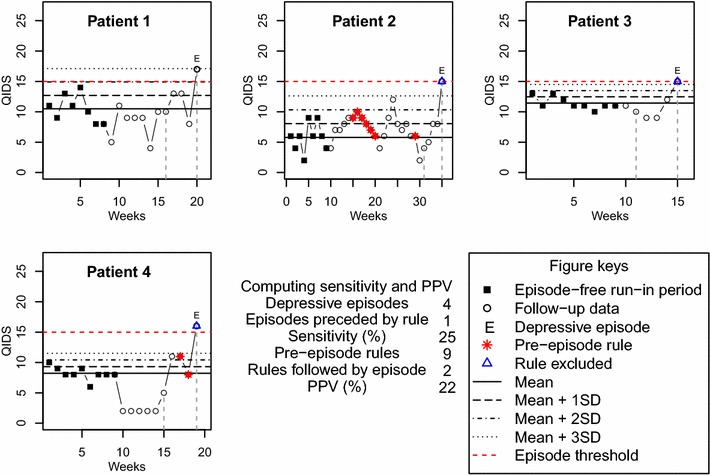
Example of sensitivity and positive predictive value calculation using personalized control charts. Calculation of the sensitivity and positive predictive value of Shewhart’s control rules to predict a depressive episode in the next 4 weeks using personalized X-bar charts for the same data used in Fig. 4

For comparison, Fig. 5 shows the corresponding calculation procedure when personalized X-bar charts are used. Given that the episode-free run-in period of each patient is used to define his/her own control chart, the control zones are different for all patients. In this particular example, both sensitivity and PPV values are smaller than the values obtained when universal control charts are used (see Fig. 4).

The sensitivity and PPV associated with run sum charts were calculated in a similar manner to the one described above, assuming that a rule was activated at the point where a special variation cause was identified.

### Main analysis

The main analysis considered X-bar charts with universal limits (i.e. based on the global mean and SD of either ASRM or QIDS scores) applied to non-imputed data from the short run-in period cohort. Missing responses were coded as − 1 and Shewhart’s control rules applied to the corresponding recoded dataset. We focused on the sensitivity and PPV of ‘any rule’ activated. That is, to calculate the sensitivity value, all rules activated within the previous 4 weeks of an episode were counted independently of the type of rule. Similarly, to calculate the PPV, for all rules activated the total number of times an episode occurred within the following 4 weeks was summed. For both metrics, the counting process was performed across all patients.

### Secondary analyses

We performed a secondary analysis evaluating the effect of the initial episode-free run-in period’s length increasing this length to 20 weeks. A different subsample was used extracted from the OXTEXT cohort, following similar criteria as for the main analysis except that only participants with 24 or more records were considered, selecting those with 20 consecutive weeks without either a severe mania or depression episode, prior to start of follow-up. Participants’ data were limited to the first severe episode as before. We will refer to this sample as the “long run-in period cohort”. There was an overlap between the short and long run-in period cohorts, as described in Fig. 1.

The impact of missing data was investigated using the last value carried forward (LVCF) imputation method, assuming that a participant’s scores remained constant until the next response was observed. All analyses were repeated using personalized X-bar charts, individual-moving range charts, and run sum charts. To explore the effect of the threshold used to define an episode on the performance of Shewhart’s control rules, the cohort extractions and, subsequently, application of universal control charts were repeated using a cutoff of 11 for QIDS [this covers moderate and severe depressive episodes (University of Pittsburgh Epidemiology Data Centre 2017)] and a cutoff of 6 for ASRM [only one point above that recommended by Altman et al. (1997)].

Manic episodes appeared to be occurring less frequently than depressive episodes; thus, we performed a post hoc analysis evaluating the sensitivity and PPV of Shewhart’s control rules to detect a manic episode within the next 8 weeks for all scenarios considered in this study.

Data extraction was carried out in Stata/SE 12.0 (StataCorp 2011). All other analyses were performed in R 3.0.1 (R Core Team 2013). Calculation of 95% confidence intervals for proportions was done using the prop. Test R function. We used the Wilcoxon rank sum test to compare the number of weeks to first episode and to first rule activation in the short- and long run-in period cohorts.

## Results

### Main analysis

An OXTEXT cohort of 219 participants with data recorded from 2001 to 2012, with median (IQR) follow-up of 60 (24–127) weeks, was available for this study. A sample of 146 participants was extracted for the main analysis following the inclusion criteria described in Fig. 1 (short run-in period cohort). Table 1 shows the baseline and episode-free run-in period characteristics for this sample. In particular, the overall episode-free run-in period mean (SD) was 2.0 (1.7) points for ASRM and 6.2 (2.5) points for QIDS.

**Table 1:** Participant and episode-free run-in period characteristics

Baseline participant characteristics	Short run-in period cohort^a^ *N* = 146	Long run-in period cohort^b^ *N* = 100
Males, *n* (%)	53 (37)	40 (41)^c^
Age, years, mean (SD)	43.4 (13.3)	44.5 (13.5)
White, *n* (%)	139 (97)	94 (96)^c^
Education, *n* (%)		
O-level/GCSE or fewer years	18 (13)	12 (12)
A-level/AS levels/Scottish Highers/HND/BTEC	24 (17)	17 (18)
Degree (includes NVQ level 5)	56 (40)	35 (36)^c^
Post-graduate degree	43 (30)	33 (34)^c^
Occupation, *n* (%)		
Disabled	1 (1)	1 (1)^c^
Housewife/homemaker	9 (6)	7 (7)^c^
Full-time student	16 (11)	9 (9)^c^
Unemployed	27 (19)	16 (17)^c^
Retired	11 (8)	9 (9)^c^
Employed	76 (54)	54 (56)^c^
Diagnosis, *n* (%)		
Bipolar disorder type I	80 (58)	60 (65)^c^
Bipolar disorder type II	53 (38)	30 (32)^c^
Bipolar disorder not otherwise specified	5 (4)	3 (3)^c^
Depression history		
Episodes, median (IQR)	10 (6–20)	10 (6–17.5)
Age of impairment, years, mean (SD)	21.7 (9.0)	23.1 (9.8)
Duration of illness, years, median (IQR)	19 (10–30)	18 (10–31)
Hospital admissions, yes, *n* (%)	63 (43)	41 (41)
Mania history		
Episodes, median (IQR)	10 (4–20)	9 (4–15)
Age of impairment, years, mean (SD)	27.2 (11.4)	28.7 (11.7)
Duration of illness, years, median (IQR)	13 (6–25)	12 (6–26)
Hospital admissions, yes, *n* (%)	46 (32)	33 (33)
Alcohol use, yes, *n* (%)	109 (86)	71 (85)^c^
Smoker, yes, *n* (%)	80 (56)	54 (55)^c^
Episode-free run-in period characteristics		
Number of prompts, mean (SD)	9 (1.1)	22.2 (2)
Percentage of missing values, mean (SD)	10 (14)	13 (14)
ASRM score across participants		
Mean score, mean (SD)	2.0 (1.6)	1.8 (1.4)
Standard deviation of score, mean (SD)	1.7 (1.0)	1.6 (0.9)
QIDS score across participants		
Mean score, mean (SD)	6.2 (2.7)	5.1 (2.4)
Standard deviation of score, mean (SD)	2.5 (1.2)	2.5 (1.0)

*n* number of observed values, *SD* standard deviation, *IQR* interquartile range

^a^Short run-in period cohort is the cohort for the main analysis defined by an episode-free run-in period 8 weeks long

^b^Long run-in period cohort is the cohort for secondary analysis defined by an episode-free run-in period 20 weeks long

^c^Percentages calculated over the total observed values

The median (IQR) waiting time from the first week after the episode-free run-in period to the first episode was 10.3 (5.3–39.6) weeks for ASRM and 10.5 (5.1–22.7) weeks for QIDS. A total of 53 (36%) participants had at least one manic episode and 67 (46%) participants had at least one depressive episode. As for the median (IQR) warning time from rule activation to episode, this was 1 (0–15) week for ASRM and 0 (0–4.5) weeks for QIDS. The average percentage of missing values was 21% (95% CI 17.8–24.2%) for ASRM and 28% (95% CI 23.9–32.1%) for QIDS, respectively.

Tables 2 and 3 show the sensitivity and PPV of Shewhart’s control rules for ASRM and QIDS, respectively, for all analyses (except for run sum charts) focusing on ‘any rule’. Results for individual and moving range charts are presented independently. Results obtained by simultaneously using both charts were similar to when only the moving range chart was used and thus omitted for simplicity. Additional files 5 and 6 contain the results for run sum charts. In the main analysis, applying universal X-bar charts on non-imputed data from the short run-in period cohort, the sensitivity of Shewhart’s control rules to detect a manic episode within the next 4 weeks was 30% (95% CI 19–45%). The PPV was 7% (95% CI 5–9%). The sensitivity of Shewhart’s control rules to detect a depressive episode within the next 4 weeks was 33% (95% CI 22–46%), whereas the corresponding PPV was 9% (95% CI 6–12%).

**Table 2:** Shewhart’s control rules sensitivity and PPV for predicting manic episodes within the next 4 weeks

	Manic episodes	Episodes preceded by any rule activation		Rules activated	Rules followed by an episode	
	*n*	*n*	Sensitivity% (95% CI)	*n*	*n*	PPV% (95% CI)
Universal X-bar charts						
Short run-in period cohort						
Original data^a^	53^a^	16^a^	30 (19, 45)^a^	439^a^	29^a^	7 (5, 9)^a^
LVCF imputed data	53	18	34 (22, 48)	755	47	6 (5, 8)
Long run-in period cohort						
Original data	25	14	56 (35, 75)	548	22	4 (3, 6)
LVCF imputed data	25	15	60 (39, 78)	958	35	4 (3, 5)
Personalized X-bar charts						
Short run-in period cohort						
Original data	53	15	28 (17, 43)	666	29	4 (3, 6)
LVCF imputed data	53	18	34 (22, 48)	1052	42	4 (3, 5)
Long run-in period cohort						
Original data	25	10	40 (22, 61)	421	15	4 (2, 6)
LVCF imputed data	25	12	48 (28, 68)	711	25	4 (2, 5)
Individual-moving range charts						
Short run-in period cohort						
Original data						
Individual chart	53	17	32 (20, 46)	944	35	4 (3, 5)
Moving range chart	53	22	42 (28, 56)	1333	49	4 (3, 5)
LVCF imputed data						
Individual chart	53	20	38 (25, 52)	1415	49	3 (3, 5)
Moving range chart	53	27	51 (37, 65)	2027	76	4 (3, 5)
Long run-in period cohort						
Original data						
Individual chart	25	10	40 (22, 61)	635	23	4 (2, 5)
Moving range chart	25	13	52 (32, 72)	1078	27	3 (2, 4)
LVCF imputed data						
Individual chart	25	11	44 (25, 65)	1012	34	3 (2, 5)
Moving range chart	25	15	60 (39, 78)	1697	44	3 (2, 3)

*PPV* positive predictive value, *CI* confidence interval, *LVCF* last value carried forward

^a^Results from main analysis

**Table 3:** Shewhart’s control rules sensitivity and PPV for predicting depressive episodes within the next 4 weeks

	Depressive episodes	Episodes preceded by any rule activation		Rules activated	Rules followed by an episode	
	*n*	*n*	Sensitivity % (95% CI)	*n*	*n*	PPV % (95% CI)
Universal X-bar charts						
Short run-in period cohort						
Original data^a^	67^a^	22^a^	33 (22, 46)^a^	395^a^	34^a^	9 (6, 12)^a^
LVCF imputed data	67	27	40 (29, 53)	777	51	7 (5, 9)
Long run-in period cohort						
Original data	32	11	34 (19, 53)	456	19	4 (3, 7)
LVCF imputed data	32	11	34 (19, 53)	924	20	2 (1, 3)
Personalized X-bar charts						
Short run-in period cohort						
Original data	67	12	18 (10, 30)	520	21	4 (3, 6)
LVCF imputed data	67	15	22 (13, 53)	848	33	4 (3, 5)
Long run-in period cohort						
Original data	32	11	34 (19, 53)	430	26	6 (4, 9)
LVCF imputed data	32	10	31 (17, 50)	749	27	4 (2, 5)
Individual-moving range charts						
Short run-in period cohort						
Original data						
Individual chart	67	13	19 (11, 31)	785	26	3 (2, 5)
Moving range chart	67	38	57 (44, 69)	3790	119	3 (3, 4)
LVCF imputed data						
Individual chart	67	17	25 (16, 38)	1222	42	3 (3, 5)
Moving range chart	67	44	66 (53, 77)	5170	160	3 (3, 4)
Long run-in period cohort						
Original data						
Individual chart	32	12	38 (22, 56)	749	30	4 (3, 6)
Moving range chart	32	22	69 (50, 83)	3246	61	2 (1, 2)
LVCF imputed data						
Individual chart	32	12	38 (22, 56)	1170	33	3 (2, 4)
Moving range chart	32	23	72 (53, 86)	4451	75	2 (1, 2)

*PPV* positive predictive value, *CI* confidence interval, *LVCF* last value carried forward

^a^Results from the main analysis

### Secondary analyses

The long run-in period cohort was formed of 100 participants. The baseline characteristics for this sample were similar to those for the short run-in period cohort (see Table 1). Twenty-five (25%) participants experienced a manic episode and 32 (32%) a depressive episode. The average percentage of missing data at follow-up was smaller than in the short run-in period cohort for both scales [3% (95% CI − 7 to 14%) less for ASRM; 4% (95% CI − 8 to 16%) less for QIDS]. Both the median time to first episode and warning time after first rule activation were higher for the ASRM scale in the long run-in period cohort than in the short run-in period cohort (34 vs 10.3 weeks, Wilcoxon rank sum test *p* value = 0.043; 13 vs 1 week, Wilcoxon rank sum test *p* value = 0.060, respectively). The correspondent values for QIDS were similar to those in the short run-in period cohort. In other words, in the study at hand, manic episodes occurred less frequently than depressive episodes.

Table 4 presents the differences between the results from the main analysis, using universal X-bar charts on non-imputed data from the short run-in period cohort, and each of the secondary analyses over non-imputed data, except for run sum charts results which use a different procedure to determine rule activations. For all secondary analyses using X-bar charts, the corresponding 95% confidence intervals indicated that the sensitivity of Shewhart’s control rules to detect manic or depressive episodes within the next 4 weeks was similar to the sensitivity obtained in the main analysis [median absolute difference = 4.7% (IQR 1.5–15.5%)]. However, the sensitivity values over the long run-in period cohort were consistently larger than in the main analysis, with the largest difference being + 26% (95% CI − 0.2 to 51.8%) when universal X-bar charts were used over ASRM data. The smallest difference was − 15% (95% CI − 30.9 to 1.1%) when personalized X-bar charts were used over short run-in period QIDS data. Individual charts performed similar to personalized X-bar charts. Moving range charts returned consistently larger sensitivity values than individual charts and universal and personalized X-bar charts. As for run sum charts, the sensitivity values were also consistently larger than for universal and personalized X-bar charts, with combination of run sum X-bar and R charts showing greater values than for each of these charts separately.

**Table 4:** Difference (95% confidence interval) between main and secondary analysis results over non-imputed data

	Non-imputed ASRM		Non-imputed QIDS	
	Sensitivity	PPV	Sensitivity	PPV
Universal X-bar charts				
Short run-in period (main analysis)	0 (0, 0)	0 (0, 0)	0 (0, 0)	0 (0, 0)
Long run-in period	25.8 (− 0.2, 51.8)	− 2.6 (− 5.6, 0.5)	1.5 (− 20.0, 23.0)	− 4.4 (− 8.0, − 0.9)
Personalized X-bar charts				
Short run-in period	− 1.9 (− 21.1, 17.3)	− 2.3 (− 5.2, 0.7)	− 14.9 (− 30.9, 1.1)	− 4.6 (− 8.0, − 1.1)
Long run-in period	9.8 (− 16.0, 35.6)	− 3.0 (− 6.2, 0.1)	1.5 (− 20.0, 23.0)	− 2.6 (− 6.4, 1.3)
Individual-moving range charts				
Short run-in period cohort				
Individual chart	1.9 (− 17.6, 21.4)	− 2.9 (− 5.7, − 0.1)	− 13.4 (− 29.6, 2.8)	− 5.3 (− 8.5, − 2.1)
Moving range chart	11.3 (− 8.7, 31.3)	− 2.9 (− 5.6, − 0.2)	53.7 (39.7, 67.8)	− 5.5 (− 8.4, − 2.5)
Long run-in period cohort				
Individual chart	9.8 (− 16.0, 35.6)	− 3.0 (− 5.9, − 0.1)	4.7 (− 17.8, 27.2)	− 4.6 (− 7.9, − 1.3)
Moving range chart	21.8 (− 4.3, 47.9)	− 4.1 (− 6.8, − 1.4)	35.9 (14.0, 57.8)	− 6.7 (− 9.7, − 3.8)

Numbers in this table can be interpreted as difference of proportions (proportion in the secondary analysis minus proportion in the main analysis) and its corresponding 95% confidence interval. A negative value indicates that the result observed in the secondary analysis was smaller than in the main analysis

*ASRM* Altman Self-Rating Mania Scale, *QIDS* Quick Inventory of Depressive Symptomatology—Self-Report, *PPV* positive predictive value

Regarding PPVs, smaller values were observed in all secondary analyses, with a median absolute difference of 4.1% (IQR 2.9–5.4%). In particular, for ASRM data, PPVs over the long run-in period cohort were smaller than in the short run-in period cohort, with a minimum difference of − 2.3% (95% CI − 5.2 to 0.7%) showed by personalized X-bar charts, and a maximum difference of − 4.1% (95% CI − 6.8 to − 1.4%) showed by moving range charts. There was not a clear pattern over QIDS, where the minimum difference was − 2.6% (95% CI − 6.4 to − 1.3%) showed by personalized X-bar charts, and the maximum difference was − 6.7% (95% CI − 9.7 to − 3.8%) showed by moving range charts. PPVs for sum rum charts were also smaller than those from the main analysis.

Increasing the time to evaluate the diagnostic accuracy of Shewhart’s control rules to predict manic episodes from 4 to 8 weeks (see Additional file 7), varying the type of chart or the length of episode-free period returned similar results to those from the main analysis. Imputing the last observed value improved the sensitivity of Shewhart’s rules, but not their PPV. The differences were non-significant, as indicated by the corresponding 95% CI is in Table 4. Reducing the cutoff to define episodes of either type had an impact on the sample size, and total number of episodes, for both cohorts. After applying the data extraction procedure described in Fig. 1, the number of patients in the short episode-free run-in period cohort changed from 146 (53 episodes) to 99 (48 episodes) in the ASRM dataset, and from 146 (67 episodes) to 96 (57 episodes) in the QIDS dataset. In the long episode-free run-in period cohort, the number of patients changed from 100 (25 episodes) to 65 (28 episodes) in the ASRM dataset, and from 100 (32 episodes) to 68 (37 episodes) in the QIDS dataset. The sensitivity of any rule when using universal control charts was slightly improved under this scenario, but not their PPV. This was true for both scales (see Additional file 8).

## Discussion

We have used control chart methodology to identify changes in mood scores in people with bipolar disorder that could indicate the emergence of a clinically important manic or depressive episode. We estimate that Shewhart’s control rules have sensitivity of 30 and 33% for manic and depressive episodes, so that approximately one-third of episodes are detected in advance. We also estimate that the positive predictive values are less than 10%, so that many ‘false positive’ alerts would occur in addition to those genuinely detecting an emerging clinical episode. Given the very significant impact major mood episodes can have on patient’s lives, being able to prevent episodes is a desirable feature of this statistical tool even though the proportion of false alarms is high. There is potential to improve the sensitivity at the expense of further false alarms, for example by using alternative control rules or different methods to calibrate the chart, as shown in Tables 2, 3 and 4.

Although approximately two-thirds of episodes would not be detected in advance by the control rules, tele-monitoring itself could detect all episodes, as defined here, at least at the moment of occurrence. Mood episodes usually last longer than a week (Solomon et al. 2010); thus, control chart methodology becomes a useful tool to provide treatment as soon as the symptoms arise. As the analysis was performed considering the scales separately, mixed episodes were not explored, nor episode length.

To the best of our knowledge, no previous attempt has been made to use control charts to aid the management of patients with bipolar disorder. We report the analysis of four types of control charts based on a normality assumption. We also explored the potential use of attribute charts for which the strong, and possibly unrealistic, assumption of the ASRM and QIDS scores following a binomial distribution is required. The binomial parameter *n* was taken as the maximum possible score (i.e. *n* = 20 for ASRM; *n* = 27 for QIDS), and the proportion parameter was estimated as the sum of scores over the episode-free run-in period divided by *n* times the episode-free run-in period length. Upper control limits were then calculated as the 68th, 95th, and 99th‰ of the corresponding binomial distribution, with the mean being equal to *n* times the estimated proportion. This type of charts was evaluated in a personalized setting only. The results were similar to those obtained for personalized X-bar charts and are mentioned here for completion (data available on request). In summary, our analysis shows that personalized and universal X-bar control limits charts have no effect on the sensitivity and PPV of Shewhart’s control rules. The sensitivity of Shewhart’s control rules is greater when using more ad hoc charts like the individual-moving range charts, but at the cost of even lower PPV than what was observed for universal and personalized X-bar charts. Moreover, these types of charts require consecutive observations, a characteristic which represents a potential practical disadvantage when monitoring bipolar patients due to the likelihood of missing data. The latter is also true for run sum charts which incorporate a much simpler procedure to identify out-of-control observations and present very high sensitivity values, although much lower PPVs than those from our main analysis. Universal X-bar charts have the advantage of providing an a priori tool for the monitoring of bipolar disorder patients that can be used straightforwardly.

The length of the episode-free run-in period used to construct the control charts also did not affect the results for either score. Missing data in the context of bipolar disorder is an important factor to consider given their potential informative nature. Due to the characteristics of bipolar disorder, patients with severe symptoms are less likely to respond when an action is required as loss of insight is a common symptom. For instance, a correlation between missing values and the standard deviation of sleep ratings has been observed (Moore et al. 2014). For these reasons, a secondary analysis evaluating the effect of missing data was performed using the LVCF method. LVCF is considered conservative and the advantages of methods like mixed models (Stevens et al. 2010) have previously been shown (Hamer and Simpson 2009). However, this method was implemented here due to its simplicity and potentially small bias (Glasziou et al. 2008). The use of mean imputation methods would have required a careful evaluation of linearity and possibly data transformation due to the nonlinear pattern observed in ASRM and QIDS scores (Moore et al. 2012a, b). The results after LVCF imputation were similar to those from the main analysis. Looking at individual rules, we observed that Rule 1 contributed the most towards the overall sensitivity and PPV reported here, whereas Rule 3 contributed the least. However, looking at the activation of any rule increases the possibility of predicting an episode by considering a greater variety of data patterns than a single rule would. For this reason, results in this paper are based on ‘any rule’ activation. An exploratory analysis of the impact of the threshold employed to define an episode of either type was performed. However, consensus on the threshold to be used to define either depressive or manic episodes should be achieved previous to constructing the control charts as suggested in this work, because the episode-free run-in period, from which the control chart defining parameters are obtained, is highly dependent on such thresholds.

The impact of medication type in the prediction performance of control chart methodology was not explored in this study. However, it is expected that as patients’ responses to different medication types vary, also the signals detected by the control charts would differ between medication types. Further investigating whether this methodology could perform better when used jointly with a specific medication type could be part of an improved treatment delivery.

The study was performed using available observational data. It is possible that selection bias is present in the results shown. External validation or simulation confirming the findings would complement this analysis. The effect of participants’ clinical and demographic characteristics on the predictive ability of Shewhart’s control rules was also not explored in this work. It is likely that taking them into account when applying control chart methodology could have resulted in a larger PPV. Busch et al. (2012) investigated the prediction accuracy for remission of random effect regression models fitted to mood scores obtained through the Montgomery-Asberg Depression Rating Scale and Young Mania Rating Scale at quarterly time points over a year. They found that prediction models that include more complete medical information have a good prediction accuracy, which is higher than those models including limited information with non-significantly different accuracy in the longer term.

Ratheesh et al. (2015) identified six instruments that predict the onset of bipolar disorder. The sensitivity and PPV of such instruments varied from 33 to 100 and from 16.7 to 72.7%, respectively. These instruments were evaluated on younger participants than those included in this study. These samples were either university students, offspring of patients with a diagnosis of bipolar disorder, or people presenting bipolar disorder symptoms at baseline. Thus, the reported sensitivity and PPVs and the corresponding values obtained in this study are not directly comparable.

Bonsall et al. (2012) focused on QIDS scores fitting different nonlinear time series to two clinically identified groups of patients (those with relatively stable or unstable mood scores) using a small sample size. The present analysis shows that control charts can equally be applied to both ASRM and QIDS scores with similar results. The nonlinear nature of the data was considered by exploring universal and personalized control limits and also by assuming a binomial distribution of the data with results also similar to the main analysis presented here.

Moore et al. (2012a, b) also used time series methodology to model QIDS scores looking at the correlation between the scale domains. They found that sleep and appetite/weight is the pair of less correlated factors and sleep variability is inversely correlated with irregularity of data. The current analysis is based on total scores which is what clinicians look at in the first instance in practice. Moreover, total scores summarize the effect of the individual domains.

Although the control charts constructed in this study are based on a sufficiently inclusive cohort, it is desirable to calculate the universal control limits based on a more representative sample. A meta-analysis to obtain more representative summary statistics for ASRM and QIDS scores could be a next step to achieve this. A randomized study of the utility of control charts in the clinical practice would provide evidence of the potential advantages of basing an intervention system on the data provided by control charts.

A further question is the possibility of defining a single control chart that combines the manic and depressive scores. We have analysed the performance of Shewhart’s control rules applied independently to depressive and manic scores. However, given the nature of bipolar disorder, there is an apparent negative interaction between these and in mixed affective states patients can experience both manic and depressive symptoms concurrently.

Finally, having a small PPV and thus a large amount of false alarms could be due to process autocorrelation. This methodological issue could be approached by exploring the use of time series and residual control charts, as suggested by Moran and Solomon (2013).

## Conclusions

Control charts are a visual display of data that can aid patients and clinicians to systematically identify when a patient should seek and receive clinical advice to prevent manic and depressive episodes. Shewhart’s control rules are designed to separate random variability from special cause changes in processes. Thus, mood scores activating any of these rules will usually tend to be beyond the control limits indicating that the patient is getting worse. In most cases, this signal alarm can occur before the mood scores reach the threshold value defining an episode as the PPV found in this work indicates. The universal control charts constructed in this study can easily be implemented in the form of a programme that prospectively monitors regular mood scores sent electronically. This potentially provides clinicians with a practical tool to manage patients with bipolar disorder.

## Additional files

**Additional file 1.** Sensitivity and PPV of individual Shewhart’s control rules for predicting manic episodes within the next four weeks using universal and personalized X-bar charts.

**Additional file 2.** Sensitivity and PPV of individual Shewhart’s control rules for predicting depressive episodes within the next four weeks using universal and personalized X-bar charts.

**Additional file 3.** Sensitivity and PPV of individual Shewhart’s control rules for predicting manic episodes within the next four weeks using individual-moving range charts.

**Additional file 4.** Sensitivity and PPV of individual Shewhart’s control rules for predicting depressive episodes within the next four weeks using individual-moving range charts.

**Additional file 5.** Sensitivity and PPV of run sum procedure for predicting manic episodes within the next four weeks.

**Additional file 6.** Sensitivity and PPV of run sum procedure for predicting depressive episodes within the next four weeks.

**Additional file 7.** Shewhart’s control rules sensitivity and PPV for predicting manic episodes within the next eight weeks.

**Additional file 8.** Sensitivity and PPV of any Shewhart’s control rule’ for predicting manic and depressive episodes within the next four weeks, when a depressive episode is defined as a QIDS score ≥ 11 and a manic episode is defined as an ASRM score ≥ 6.

## Abbreviations

ASRM: Altman Self-Rating Mania Scale
CI: confidence interval
DSM-IV: Diagnostic and Statistical Manual of Mental Disorders, 4th edition
IQR: interquartile range
LVCF: last value carried forward
OXTEXT: study about the benefit of self-monitoring in people with bipolar disorder using the True Colours self-management system (https://oxfordhealth.truecolours.nhs.uk/www/en/)
PPV: positive predictive value
QIDS: quick inventory of depressive symptomatology self-report
SD: standard deviation
